# Patterns of biomarkers for three phenotype profiles of persisting specific learning disabilities during middle childhood and early adolescence: A preliminary study

**Published:** 2017-06-19

**Authors:** Robert D. Abbott, Wendy H. Raskind, Mark Matsushita, Nathan D. Price, Todd Richards, Virginia W. Berninger

**Affiliations:** 1 University of Washington, Quantitative Studies and Measurement, USA; 2 University of Washington, Medicine, USA; 3 University of Washington, Psychiatry and Behavioral Sciences, USA; 4 Institute for Systems Biology, USA; 5 University of Washington, Bioengineering, Computer Science & Engineering, Molecular & Cellular Biology, USA; 6 University of Washington, Integrated Brain Imaging Center and Radiology, USA; 7 University of Washington, Learning Sciences and Human Development, USA

**Keywords:** dysgraphia, dyslexia, OWL LD, genetic biomarkers, hallmark phenotypes, cascading levels of language, translation science

## Abstract

Students without specific learning disabilities [SLDs] [*n*=18] and with one of three persisting SLDs in written language despite early and current specialized instruction—Dysgraphia [*n*=21], Dyslexia [*n*=40], or oral and written language learning disability OWL LD [*n*=14]— in grades 4 to 9 [*N*=56 boys, 38 girls] completed behavioral phenotyping assessment and gave a small blood or saliva sample. Molecular analyses informed by current cross-site research on gene candidates for learning disabilities identified associations between molecular genetic markers and the two defining behavioral phenotypes for each SLDs-WL; dysgraphia [impaired writing alphabet from memory for rs3743204 and sentence copying in best handwriting for rs79382 both in DYX1C1], dyslexia [impaired silent word reading/decoding rate for rs4535189 in DCDC2 and impaired spelling/encoding for rs374205 in DYX1C1], and OWL LD [impaired aural syntax comprehension for rs807701 and oral syntax construction for rs807701 both in DYX1C1]. Implications of these identified associations between molecular markers for alleles for different sites within two gene candidates [and mostly one] and hallmark phenotypes are discussed for translation science [application to practice] and neuroimaging that has identified contrasting brain bases for each of the three SLDs.

## Introduction

A variety of methodologies, ranging from twin studies [1,2] to genetic linkage analyses [3] to aggregation analyses of a phenotype [behavioral marker showing evidence of cross-generational genetic bases] and segregation analyses [identification of potential genetic patterns of transmission] [4] to genome wide scanning to identify gene candidates [5] have been applied to demonstrate the genetic bases of specific learning disabilities in otherwise typically developing children and youth. More recently genetics researchers have begun to investigate the molecular mechanisms of genetic transcription [protein coding and non-protein coding] and translation [generating amino acids from mRNA messages] that are related to, but not identical with, the gene candidates. The latter kind of research involves laboratory analyses to identify downstream effects of alleles [DNA variations between individuals that may or not have a detectable effect on phenotypes].

A major problem for all of these genetic studies is that researchers have not reached consensus on how to define specific learning disabilities. Ascertaining simply on the basis of poor reading, for example, can be problematic because poor reading occurs for many different reasons. For example, dyslexia and specific language impairment [SLI], both of which may interfere with reading acquisition, are not the same disorder at the behavioral level [6]. It is also likely the case that they are also not identical at the molecular genetics level.

The purpose of this preliminary study was, therefore, to extend prior behavioral [7] and brain research [8–11] showing differences in profiles [patterns of expression] in specific learning disabilities in written language [SLDs-WL] that persist beyond the early grades despite prior and current specialized instruction. For example, when entered last in sequential entry multiple regression, profiles for dysgraphia [impaired handwriting], dyslexia [impaired word reading and spelling], and oral and written language learning disability [OWL LD, impaired oral and written syntax] contributed unique variance to reading and writing outcomes [7]. For the same word-specific spelling phenotype contrasting fMRI brain connectivity was observed among those with dysgraphia, dyslexia, or OWL LD, who differed in behavioral expression at cascading levels of language [subword letter production, word reading and spelling, and syntax comprehension and construction] [8].

The hypothesis was tested that the defining hallmark phenotypes for these three contrasting SLD-WL profiles might also differ in molecular genetic markers, that is, alleles. Although alleles or variations at the molecular level may be related to gene candidates in the sequenced human genome, they are not identical with those gene candidates, just as phenotypes are behavioral markers but not necessarily identical with the underlying genetic variables. Nevertheless there may be associations between profiles of phenotypes that differentiate persisting SLDs-WL and profiles of alleles that are molecular markers of their genetic bases.

Moreover, studying such molecular genetic markers may supplement large scale genome- wide sequencing studies and inform future research on downstream epigenetic effects of students’ response to developmentally appropriate written language instruction or other interventions in or out of the school environment. Epigenetic changes are chemical alterations to the DNA that do not change the DNA sequence that was inherited from a parent, but that may modify gene expression [12]. Molecular biomarkers may be important mediating variables in epigenetic responses to instruction for contrasting SLDs- WL. It is first necessary, however, to identify which molecular markers may be related to specific phenotype profiles.

As such the goal is not to add to basic science understanding of genetic mechanisms in the genome but rather conduct a preliminary study relevant to the emerging field of *translation science for applying basic genetics research to clinical and educational practice.*

Such translation science could have four potential benefits: [a] policy makers can understand that teachers, parents, and students should not be blamed for failure of children with genetically based SLDs to respond to instruction; [b] interdisciplinary teams in schools can be better prepared to explain to parents and teachers why some students continue to struggle in the upper grades despite considerable early intervention based on the findings of the National Reading Panel [13]; [c] affected students do not think they are “damaged goods,” and should not have children when they grow up, as some parents reported to authors; and [d] genetic and molecular “barriers” to learning can be ameliorated by educational approaches that are specially designed to overcome those “barriers”.

### Challenges in studying associations between molecular markers and phenotype markers heterogeneity

Heterogeneity has been documented at both genetic and behavioral levels of analyses [14,15]. Thus, the ongoing controversies about how to define SLDs in diagnostic manuals, state departments of education for regulations regarding eligibility criteria for special education services, and policies for accommodations for disabilities are understandable. Nevertheless, given the large number of affected children and youth at some time in their schooling, studies seeking to define such heterogeneity within and between the molecular and behavioral levels are warranted. Epidemiological studies in programmatic research at the Mayo Clinic showed that SLDs impair learning to write, read, and use heard and spoken language in one in five school age children and youth [16–18]. Many are affected but not necessarily in the same way.

### Developmental changes in neurogenetic mechanisms and environmental variables

Another challenge has been the normal changes in developing learners, related, in part, to neurogenetic mechanisms operating from conception [19] to early childhood [neuronal pruning about second year of life] [20] to middle childhood to adolescence and even adulthood transitions [increasing myelination of neural networks] [20]. Such developmental change is also due in part, to the changing nature and requirements of curriculum across elementary, secondary, and postsecondary education [21]. Although considerable progress has been made in evidence-based screening and intervening in the early grades to prevent or at least reduce the severity of SLDs in language learning [22,23], some students have persisting SLDs beyond the early grades in language skills, which continue to be or become relevant in the upper grades [24,25].

To begin with, although most reading instruction in the early elementary grades emphasizes oral reading, in the upper elementary grades and thereafter most instruction and assignments involve silent rather than oral reading. Yet silent reading skills are not necessarily taught explicitly in the upper grades; and oral reading may not transfer in a simple way to silent reading, which relies greatly on internal processing without the support of oral production and the resulting aural feedback from hearing oneself read aloud [26].

Moreover, in the US handwriting is typically taught only in the early grades and increasingly only in kindergarten and first grade because of common core standards; yet many written assignments and tests are completed at school without use of technology to support writing. Consequently, students with handwriting difficulties may continue to struggle with learning to spell written words [27] and thus complete written assignments in the upper elementary grades and beyond because they cannot form letters legibly and automatically, spell words, and sustain written language output over time [28].

In addition, not all schools provide systematic spelling instruction across the elementary grades because they mistakenly assume that it is not necessary when students can rely on spell checks in their word processing programs. In reality, many, if not most, elementary classrooms still do not integrate computers with their literacy instruction in general education. Also spell check only offers a menu of options from which to choose the correct spelling—so knowledge of word-specific spelling [29,30] that links orthography, phonology, morphology [31], and semantics is still needed to choose the correct spelling that fits the sentence context. Also, research has shown that children with oral reading problems in the early grades often have persisting spelling problems in the upper elementary grades and beyond [32] which interfere with their written composition [33].

Despite the myth that children learn oral language before coming to school, it is still the case that oral language learning continues to be relevant and to develop during the school years. Students have to learn to listen to teachers’ instructional talk, and express their ideas orally through discussions with classmates and in response to teachers’ questions. The academic register of talk used at school in the formal learning environment differs in many ways from the informal register used in conversation outside school and on the playground and lunchroom. In fact, some students have SLDs in oral language that emerge at the time of first words or combining multiple words during the preschool years, but continue to interfere during the school years with their learning from oral language others use and using oral language to communicate with others and express their own thinking [34–36]. However, current policies and procedures often do not differentiate between those students who have oral as well as written language learning difficulties beginning in the preschool years and those who have problems in word reading/decoding and word spelling/encoding that emerge during the school years, usually beginning in kindergarten when letter writing, letter naming, and letter-sound correspondences are first formally taught. As a result, not all the students with OWL LD are identified or provided with specialized instruction tailored to their oral as well as written language learning disabilities [37].

### Importance of studying molecular and behavioral markers despite challenges

Yet despite these curriculum issues related to instruction provided in the upper grades, many students develop grade-appropriate silent reading, handwriting, spelling, and oral language problems. Thus, the likelihood that genetics also plays a role in the persisting handwriting, silent reading, word spelling, and oral language disabilities during middle childhood and early adolescence deserves research attention. In sum, both genetic and environmental sources of change, independently and interactively, across time may contribute to the complexity of biological and behavioral mechanisms and their interrelationships in oral and written language learning. Despite the challenges, research on the relationships between molecular genetic markers and behavioral markers of SLDs-WL is warranted.

### Lack of comparability in defining sample characteristics

Variation in how researchers ascertain their samples, including inclusion criteria and thus the resulting particular mix of the various kinds of written and/or oral language learning difficulties represented in their samples, continues to pose problems for generalizing research findings across research groups no matter how large a sample may be. For example, assigning all participants with spelling disabilities to the diagnostic category of dyslexia does not take into account that some developing children and youth have handwriting problems which result in spelling problems but do not have reading problems, whereas others have both word reading/decoding and spelling/encoding problems. So spelling problems alone may not adequately differentiate among the phenotypes and their related genetic alleles. Likewise, some may have reading problems related to identifying real words or pseudowords, which may interfere with reading comprehension, but others may have significant reading comprehension problems without significant word decoding problems or may have reading comprehension problems that are not directly related to their word decoding problems—other skills at the syntax [38] and/or text levels of heard aural language or produced oral language may also be contributing to their reading comprehension problems.

### Operationalizing diagnostic profiles [patterns] in the current research

The behavioral phenotype profiles were based on assessment measures previously validated in behavioral research across disciplines [37] as well as recent interdisciplinary behavioral [7] and brain [8] research. For molecular markers we used alleles based on gene candidates for learning disabilities that have been identified in research across countries [4]. For example, see [39] for these findings: [a] dyslexia candidate genes DYX1C1, DCDC2, and KIAA0319 have been associated with dyslexia, neuronal migration, and ciliary function; [b] specific alleles at the polymorphic sites rs3743204 in DYX1C1, rs793842 in DCDC2, and rs6935076 in KIAA0319 have been linked to variability of left temporoparietal white matter volume connecting the middle temporal cortex to the angular and supramarginal gyri; [c] alleles of rs793842 in DCDC2 have been associated with the thickness of left angular and supramarginal gyri; and [d] the left lateral occipital cortex has been shown to be significantly thicker for T-allele carriers, who also had lower white matter volume and lower reading comprehension scores. Thus, these allele-phenotype relationships may provide insights for designing future research on gene-brain relationships of the students with specific kinds of persisting SLDs.

### Measurement issues

A further challenge is related to differences in measurement scales used for behavioral phenotypes on standardized tests and the data that molecular genetic analyses yield. Behavioral phenotypes are *continuous variables* for units of written or oral language that are relevant to assessing the various levels [units] of language that may be impaired in SLDs-WLs based on norms for age or grade peers; they identify where in the normal distribution for age or grade an individual’s score falls. In contrast, alleles are a single nucleotide [A: adenine, T: thymine, C: cytosine, or G: guanine], that is, nominal variables rather than continuously distributed variables that locate a skill along a normal distribution for age or grade peers. It follows that identifying relationships between specific phenotypes and specific alleles will require statistical methods that combine approaches for analyzing both differences in means of continuous quantitative phenotype variables and related associations with nominal allele genetic variables for classifying individuals. Such methods are not the same as those used in genome wide sequencing to identify gene candidates.

### Research approach and hypothesis

To begin with, this preliminary study was grounded in programmatic interdisciplinary research and not conventional genetics or psychological/educational assessment methods. Rather the goal was to validate a proof of concept for this interdisciplinary approach for assessing associations between molecular markers and behavioral phenotypes, which may have translation science applications. These associations, which involved distributed and nominal measures, were tested based on students with and without specific kinds of SLDs-WL but analyzed for the sample as a whole. For participants with SLDs- WL there had to be evidence both on normed behavioral measures and current and past history that these were persisting problems despite earlier and current intervention, However, we also took into account when the SLDs were first observed—during the preschool years or only after formal instruction began in school in kindergarten or first grade. Allele-phenotype relationships were analyzed for the two evidence- based hallmark, developmentally appropriate phenotypes for each of three profiles defined on the basis of impairment in cascading levels of language—subword letters, words, and syntax. We tested the hypothesis that associations between the molecular genetic markers [alleles] and behavioral markers [phenotypes] could be identified for the two hallmark phenotypes for each of the three SLDs-WL studied.

## Methods

### Ascertainment of participants

Recruitment, sampling of blood or saliva for DNA, and behavioral assessment were performed under protocols approved by the Institutional Review Board for Research and conducted in manner that complied with the ethical principles of the American Psychological Association. Participants were recruited through flyers distributed through local schools and the university, but some parents heard about the study through word of mouth of other parents who had participated or by educators who referred them because of student failure to respond to intervention. The flyers announced an opportunity to participate in research for students in grades 4 to 9 with and without SLDs in oral and written language.

When parents contacted the research team, a screening interview with scripted questions was conducted over the phone to rule out other conditions that could account for the learning problems other than an SLD; that is, the reading or writing disabilities had to be specific in otherwise typically developing students and not related to other neurogenetic, medical conditions, toxins, or injuries. Such conditions for exclusion included pervasive developmental disability, neurogenetic disorders like fragile-X, neurofibromatosis or phenylketonuria, sensory disorders like significant hearing loss or visual impairment, motor disorders like cerebral palsy or muscular dystrophy, spinal cord or brain injuries, substance abuse, and other medical conditions like epilepsy. Attention deficit/hyperactivity syndrome [ADHD] was not an exclusion criterion.

If responses to the interview questions indicated the child would probably qualify and was typically developing with or without an SLD involving oral and/or written language learning, and the parent granted informed consent and the child assent, then a comprehensive assessment was scheduled at the university where the research was conducted. If the child and parent gave permission for participation in genetic studies a sample of blood or saliva was collected for DNA extraction and laboratory molecular genetic analyses.

### Phenotype assessment

The assessment included measures of handwriting [from memory in alphabetic order and copying from models of letters in written words], silent word reading fluency, word-specific spelling, listening comprehension, oral syntax construction, and verbal comprehension [orally explaining concepts]. While children were tested, their parents completed questionnaires about the child’s developmental history, educational history, and family history. Both test scores and information in these questionnaires were used in assigning students to diagnostic groups. Of particular interest was [a] when the problems first emerged—early in language development during the preschool years, as in OWL LD [40,41] or early in the school years when letter production and oral reading are emphasized, as in dysgraphia and dyslexia [42]; and [b] whether they had persisted during middle childhood [upper elementary grades] and early adolescence [middle school grades], despite earlier intervention during the preschool or school years, when curriculum requirements change [current study].

The test battery included the following measures for hallmark phenotypes based on prior research [8]. The first two measures assessed handwriting, which is impaired in dysgraphia. The third and fourth measures assessed silent word reading and written spelling, both of which are impaired in dyslexia. The fifth and sixth measures assessed oral language—listening comprehension and oral expression, which are impaired in OWL LD. The seventh measure assessed verbal comprehension [translation of cognitions into oral language] to evaluate if the participants were typically developing language learners within the normal range.

#### Automatic alphabet letter writing from memory:

Children are asked to handwrite in manuscript [unjoined letters] the lower case letters of the alphabet from memory legibly so others can identify the letters and as quickly as possible without sacrificing legibility. The raw score is the number of letters that are legible and in correct alphabetic order during first 15 seconds, which research has shown is an index of automatic compared to controlled processing [43] and may be impaired in those with dysgraphia [8] and/or dyslexia [44]. The raw score is converted to a z-score [*M*=0, *SD*=1], based on research norms for grade [inter-rater reliability .97], from a large sample of students with and without SLDs.

#### Copying letters in words in sentences.

On the *Detailed Assessment of Speed of Handwriting [DASH] Best* [45], the task is to copy a sentence with all the letters of the alphabet in one’s best writing. Students can choose to use their usual writing—manuscript [unconnected] or cursive [connected] or a combination of these. The scaled score [*M*=10, *SD*=3], derived from transformation of the raw score, is based on legibility for single letters within the time limits [interrater reliability .99]. In the current study, two testers reviewed all the scored handwritten measures to reach consensus on scoring.

#### Silent word reading:

On the *Test of Silent Word Reading Fluency [TOSWRF]* [46] [test-retest reliability is .92] the task is to mark the word boundaries in a series of letters arranged in rows. The score is the number of correctly detected and marked word boundaries in 3 minutes. The raw scores are converted to standard scores for age [*M*=100, *SD*=15].

#### Word-specific spelling:

For the *Letter-Choice* subtest [test-retest reliability .84 to .88] of the *Test of Orthographic Competence [TOC]* [47], the task is to choose a letter in a set of four provided letters to fill in the blank in a letter series to create a correctly spelled real word [word-specific spelling]. Raw scores are transformed into a scaled score [*M*=10, *SD*=3].

#### Listening comprehension:

*WJ III Oral Comprehension* [48] *Oral Comprehension* [test-retest reliability .88], which is an aural cloze task, requires supplying a word orally during pause in unfolding oral text. Raw scores are transformed into a standard score [*M*=100, *SD*=15].

#### Construction of oral syntactic structures from three provided words [pictured and spoken]:

The *Clinical Evaluation of Language Function 4*^*th*^ *Edition CELF IV* [49] *Formulated Sentences* [test-retest reliability .62 to .71] is a task that requires constructing an oral sentence from three provided words. Raw scores are transformed into scaled scores [*M*=10 and *SD=* 3].

#### Translating cognitions through oral language

*[heard language through ear and spoken language through the mouth]*. Raw scores on the *Wechsler Intelligence Scale for Children, 4*^*th*^ *Edition [WISC IV]* [50] *Similarities, Vocabulary, and Comprehension* subtests were converted to scaled scores, which were combined to obtain a standard score [*M*=100, *SD*=15] for a Verbal Comprehension Index score [test-retest reliability .93 to .95]. This measure was given to document that the student was typically developing regardless of the oral and/or written language learning problems [at least a standard score of 80, which is 1 1/3 SD below the population mean] or might be twice exceptional [superior or better on Verbal Comprehension Index despite an SLD].

#### Assignment to SLD or control groups:

For the tests scores, the criteria that follow were the minimum criteria for qualifying for a diagnostic group [dysgraphia, dyslexia, OWL LD, or no SLDs-WL], but most who qualified fell substantially below the criteria. Participants had to quality for SLD diagnostic group assignment on at least two measures. All had reported history of past and current struggle with the relevant language skills for the SLD group to which they were assigned. These criteria were as follows.

To qualify for the *Dysgraphia Group,* the participant had to score below −2/3 SD [25^th^ %tile] on two or more handwriting measures and have parental report of a history of past and current handwriting difficulties, but no current or past reading problems or preschool oral language problems. To qualify for the *Dyslexia Group*, the participant had to score below −2/3 SD [25^th^ %tile] *or* below population mean and at least 1 SD [15 standard score points] below Verbal Comprehension Index on silent word reading fluency and word-specific spelling; no current syntax level listening comprehension or oral expression difficulties; and parental report of a history of past and current word decoding/real word reading [oral and/or silent] and/or spelling problems but no preschool or current oral or aural language problems. About three fourths of the participants assigned to the dyslexia group met these criteria, but some who had superior cognitive scores [i.e., twice exceptional] fell below the population mean and at least one standard deviation below their Verbal Comprehension Index score and had a history of struggling in learning to read/decode words and spell/encode words [51,52]. To qualify for the *OWL LD Group*, the participant had to score below −2/3 SD on two measures of syntax-level language skills—*WJ III Oral Comprehension* and *CELF 4 Formulated Sentences* and parental report of preschool history of oral language problems and persisting problems in listening comprehension, reading comprehension, oral expression and/or written expression. To qualify for the *Typical Language Learning Control Group*, participants had to score at or above standard score of −2/3 SD on all measures of language by ear, by mouth, by eye, and by hand and have no reported past or current history of oral and/or written language learning problems.

#### Comparison of diagnostic groups on hallmark behavioral phenotypes:

Means on each hallmark phenotype measure were calculated for the dysgraphia, dyslexia, or OWL LD and control groups. ANOVA was first used to evaluate if main effects were statistically significantly different for groups; and if so, then pairwise comparison of the groups was done with *t*-tests. The purpose of these analyses was to determine if diagnostic groups differed at the behavioral level before examining potential allele-phenotype associations that transcend assignment to a diagnostic group for the language learners in general in grades 4 to 9.

#### Participant characteristics:

The total sample [*N*=94] consisted of 56 boys and 38 girls. Table 1 summarizes the descriptive statistics of their performance on the test measures used for assignment to diagnostic groups: typical language learning control group [*n*=18], dysgraphia group [*n*=21], dyslexia group [*n*=40], and OWL LD group [*n*=14]. Based on self-reported ethnicity the majority [*n*=86 mothers and 78 fathers] were of European or Middle Eastern ancestry, 1 mother and 1 father were African American, 2 mothers and 5 fathers were Asian American, 3 mothers and 2 fathers were Hispanic, 1 mother and 1 father were Pacific Islander, and 2 mothers and 2 fathers were other/ non-specified. Parents’ self-reported educational levels ranged from less than high school [1 mother and 1 father] to high school graduate [2 mothers and 3 fathers] to more than high school but less than college [5 mothers and 12 fathers] to college [40 mothers and 31 fathers]; some did not report educational levels [8 mothers and 12 fathers].

### Analyzing biomarkers [Polymorphisms]

Selecting sites to analyze: Candidate genes and polymorphisms within them were selected on the basis of prior research support for them. The sites evaluated were single nucleotide polymorphisms [SNPs] rs3743205, rs570809907, and rs3743204 in DYX1C1, rs807701, rs793862, and rs793842 in DCDC2, and rs4504469, rs9461045 and rs6935076 in KIAA0319, and rs6803202 and rs4535189 in ROBO1.

DYX1 rs3743205 [−3G>A] created an ELK1 binding site [53], TFII-I + PARP1 + SFPQ/PSF complex binds to −3G, whereas binding to −3A was weak [54]. After co-transfection of allele-specific constructs into a neuroblastoma cell line, expression of the −3G allele was higher than that of the −3A allele [54],

−3A was associated with decreased accuracy of reading [55], and methylation of the −3G allele markedly decreased expression but had no effect on the −3A allele [56]. DYX1 rs57809907 [c.1249G>T, p.E417X] truncated the last 4 amino acids of the protein [53].

The volume of temporoparietal white matter was greater with the GG genotype than either the GT or TT genotypes of DYX1 rs3743204 [G>T] [57] and the major allele, G, was associated with poorer irregular word and nonword reading in the general population [58,59]. For DCDC2, rs793842 was significantly associated with the thickness of left angular and supramarginal gyri as well as the left lateral occipital cortex. The cortex was significantly thicker and the white matter volume was less in carriers for the T allele, which was also found to be associated with lower reading comprehension scores [39]. The A allele of rs793862 was associated with dyslexia in a German study, especially in those with a “nondysphonetic” subtype characterized by problems with discrimination of written words and not with problems in differentiation and synthesis of sounds [60,61]. The minor allele, T, of rs4504469 in KIAA0319 resulted in a nonsynonymous substitution in exon 4 of threonine for alanine at residue 266. The minor allele of rs9461045 reduced expression of KIAA0319 possibly through creation of a binding site for the transcriptional silencer OCT-1 [62]. Decreased white matter volume in left temporoparietal regions was observed for the TT genotype of rs6935076 in KIAA0319 [39,57].

#### Laboratory molecular analyses of alleles:

SNP genotypes were obtained with TaqMan methodology using an ABI 7500 Real-Time PCR system and ABI Sequence Detection Software. The DRD4 exon 3 VNTR that encodes the third intracellular loop of the receptor comprises between 2 and 11 repeats of 48 bp. Allele sizes were determined by fragment analysis on an ABI Prism 3130XL Genetic Analyzer, using published primer sequences and conditions with slight modifications [63]. The VNTR in the 3´ untranslated region of the dopamine transporter SLC6A3 gene, also known as DAT1, ranges from 3 to 11 copies of a 40bp repeat [64,65].

### Behavioral statistical analyses of allele-phenotype relationships

For each of six hallmark phenotype measures linked to a particular diagnosis [for *dysgraphia,* alphabet writing from memory and copy in best handwriting; for *dyslexia,* silent word reading fluency and adding a letter to create word-specific spelling; and for *OWL LD* listening comprehension and oral expression], separate ANOVAs evaluated whether the mean values on the hallmark phenotypes [continuously distributed quantitative values] differed by allele at these sites (The nonsignificant results are available upon request from the first author.) For significant main effects for hallmark phenotypes with three or more chemical combinations, follow-up Tukey LSD tests were then used to pairwise compare the chemical combinations [alleles] to evaluate if they were significantly different in the magnitude of the associated phenotype.

## Results

### Phenotype differences among groups

First, results of comparing each of the SLD diagnostic groups to the typical language learning controls on the behavioral phenotypes are reported. Table 1 provides means and SDs for each of the hallmark phenotype measures in each group. Insert Table 1 about here.

Next, results for comparing each of the SLD groups to each other across levels of language [increasing written language unit size—from subword letter to word to multi-word] on the behavioral phenotypes are reported.

#### Comparison of SLD groups with typical control group:

On both *Alphabet 15* and *Copy Best,* the control group had higher mean scores than the dysgraphia group. On both *TOSWRF* silent word reading fluency and *TOC Letter Choice*, the control group had higher mean scores than the dyslexia group. On *WJ III Oral Comprehension* and *CELF 4 Formulated Sentences,* the control group had higher mean scores than the OWL LD group.

#### Comparisons of SLD groups with each other:

The dysgraphia group with subword letter impairment was compared to the control group, the dyslexia group with word impairment was compared to the dysgraphia group, and the OWL LD with syntax impairment was compared to the dyslexia group with word impairment. On the following measures the dysgraphic group scored lower than the typical control group: *Alphabet 15 F*[1,37]=13.41, *p*=.001 and *Copy Fast F*[1,32]= 23.30, *p*=.001 [both phenotypes unique to dysgraphia]. On the following measures the dyslexic group scored lower than the dysgraphic group: TOSWRF, *F*[1,56]=9.99, *p*=.003, and *TOC Letter Choice F*[1,47]= 13.93, *p*=.001 [both phenotypes unique to dyslexia]. On the following measures the OWL LD group scored lower than the dyslexic group: *WJ III Oral Comprehension, F*[1,52]=35.77, *p*=.001, and *CELF 4 Formulated Sentences F*[1,53] = 71.32, *p*=.001 [both phenotypes unique to OWL LD]. These findings are consistent with the cascading level of language model according to which dysgraphia is associated with sub-word level impairment, dyslexia is associated with word-level impairment, and OWL LD is associated with syntax-level impairment [8].

### Alleles related to hallmark phenotypes for each SLD diagnostic group

Next, the mean quantitative score for each continuously distributed behavioral phenotype was evaluated in relationship to the pattern of alleles at a particular polymorphism in a candidate gene. Significant relationships are reported for the two hallmark phenotypes for each SLD. The nonsignificant results are available upon request from the first author.

#### Significant allele-phenotype relationships for dysgraphia:

Significant allele-phenotype relationships were found for both hallmark phenotypes for impaired handwriting—legible automatic alphabet writing from memory and copying sentence with all alphabet letters in one’s best handwriting. These were found for two different alleles involving different chemical combinations of base chemicals within a common gene site.

For *rs3743204* in DYX1C1, there were significant differences in alphabet 15 associated with this allele variation: *F*[2,91]= 3.98, *p*=.022: GG [*N*= 57, *M*= −1.19; *SD*=0.76] GT [*N*=33; *M*=−1.46; *SD*=0.71] TT [*N*=4; *M*=−.41, *SD*=.77]. By Pairwise Tukey test there was no significant difference in mean score on phenotype associated with GG and GT, *p*=.111; but GT was significantly worse than TT, *p*=.009; and GG was significantly worse than TT, *p*=.044. On contrast coding, GT and GG were significantly worse than TT, *t*[91]=2.407, *p*=.018. Thus, absence of two Ts [homozygosity for G] at this site appears to be a risk factor for this hallmark phenotype for dysgraphia—legible, automatic alphabet letter writing from memory. Those with TT performed better.

For *rs793842,* also in DYX1C1, there were significant differences in *DASH Copy Best* associated with the allele pattern: *F*[2,66]= 3.64, *p*=.032; CT [*N*=33; *M*=10.82; *SD*=3.03]; TT[*N*=10; *M*=8.80; *SD*=3.58]; and CC[*N*=26; *M*=8.69; *SD*=3.33]. By pairwise Tukey test: CT was associated with a significantly better score than CC, *p*=.014, but scores associated with CT were not significantly different from those with TT, *p*=.088, and scores with TT were not significantly different from CC, *p*=.929. Thus, homozygosity or heterozygosity for the T allele may facilitate better performance on the *Copy Best* test.

#### Significant allele-phenotype allele relationships for dyslexia:

Significant phenotype-allele relationships were found for both hallmark phenotypes for dyslexia—impaired silent word reading fluency on *TOSWRF* and impaired word-specific spelling on *TOC Letter Choice*. For silent word reading fluency, these were found within the same polymorphism but for different alleles. However, for word- specific spelling, the allele-phenotype relationship was found for a different polymorphism [same as for handwriting] than for silent word reading fluency, but it involved the same genotypes as *DASH Copy Best,* a handwriting task for copying words as opposed to retrieving single letters in alphabet from memory.

For *rs4535189,* in DCDC2, individuals with genotype AG had significantly higher *TOSWRF scores* [*M*=96.5, SD=10.62] than individuals with GG [*M*=90.59, *SD*=8.99], *F*[1,59]=5.98, *p*=.017. Thus, homozygosity for the G allele appears to be a risk factor for silent word reading fluency. For rs6803202, also in DCDC2, individuals with genotype CT [*M*=96.8, *SD*=10.62] had significantly higher *TOSWRF* scores than individuals with TT [*M*=90.21, *SD*=8.54], *F*[1,60]=7.14, *p*=.010. Thus, in this site homozygosity for the T allele appears to be a risk factor for silent word reading fluency.

For *rs374205,* in DYX1C1, there was a significant difference between the CC and CT genotypes on the *TOC Letter choice s*pelling measure; *F*[1,72]= 3.97, *p*=.011. Mean score for genotype CC [*N*=62, *M*=8.45, *SD*=2.95] was higher than the mean score for CT [*N*=12, *M*=6.42, *SD*=2.47]. This finding for word-specific spelling is of interest for three reasons. First, in contrast to silent word reading fluency, the risk factor appears to be heterozygosity rather than homozygosity. Second, as was the case for silent word reading fluency, presence of T may be a risk factor for word-specific spelling, although in this case even a single T confers this risk compared to two Ts for silent word reading fluency. Third, for the measure of spelling, which is learned through output in handwriting, the gene site [if not the allele itself] was the same as for impaired handwriting, whereas for the measure for silent reading, which is learned through input through eyes, involved a different gene site than handwriting did.

#### Significant allele-phenotype relationships for OWL LD:

*For rs807701* in DYX1C1, there were significant differences for Oral Comprehension associated with the genetic variation: *F*[2,66]= 3.57, *p*=.034 AG [*N*=25; *M*=114.28; *SD*=11.76] GG [*N*=8; *M*=111.75; *SD*=7.25] AA [*N*=36; *M*=105.78; *SD*=−13.73]. By pairwise Tukey test, genotype AA is a greater risk factor than AG, *p*=.011. Performance associated with GG is not significantly different from that of AA, *p*=.225 or AG, *p*=.619. For *rs807701,* which is also in DYX1C1, there were significant genetic variations associated with *CELF 4 Formulated Sentences, F*[2,72]= 3.16, *p*=.048: AG [*N*=27; *M*=11.19; *SD=* 3.00], AA[*N*=38; *M*=9.66; *SD*=3.41], and GG [*M*=10; *M*=8.40; *SD*=3.57]. By Pairwise Tukey: AG is not significantly different from AA, *p*=.068; AG is significantly greater than GG, *p*=.025; and AA is not significantly different from GG, *p*=.284.

Thus, for OWL LD genotypes at two polymorphic locations within this gene appear to be related to the aural comprehension [AA] and oral expression [GG] phenotypes that characterize this SLD. If not taken into account in instruction, reading comprehension and written expression problems related to syntax may occur.

#### Analyses with only European American participants:

Because population stratification - the phenomenon of different relative frequencies of alleles in different ethnic populations - can confound association studies, the analyses were repeated with only the children of European American descent included. The results, which are available upon request from the first author, replicated those when all ethnicities were included in the analyses of phenotype-allele relationships.

## Discussion

### Significance of current study for genetics research

Based on the many reported relationships between gene candidates and phenotypes and their unreliability across studies in the research literature many genetics researchers have concluded that the gene- behavior relationships are heterogeneous and require very large population samples to identify reliably. An alternative approach less employed but promising nevertheless is to [a] study these relationships using well defined phenotypes based on profiles of co-occurring phenotypes rather than a single phenotype in isolation, [b] phenotypes defined based on normed measures for age or grade peers that can be interpreted across development rather than raw scores; and [c] phenotypes that take into account age when first behavioral signs emerged and past and current history of phenotype expression. The heterogeneity and unreliability may also result, in part, from failure of researchers to ascertain using the same procedures and developmentally and diagnostically appropriate criteria for inclusion. In this current study we report the results of a study designed for purposes of translation science which used a smaller sample but well defined inclusion criteria with multi-criteria for the nature of the disability studied in a specific stage of development [middle childhood and early adolescence]. The results can only be generalized and applied to practice for those criteria and that development time period. Such an approach might supplement rather than replace large population studies not using exactly the same acquisition procedures and phenotype criteria.

### Evidence for contrasting allele-phenotype associations across SLDs-WL

The results of this initial study of allele-phenotype relationships in oral and/or written language SLDs hold promise for translation science, if they replicate in additional samples ascertained using comparable inclusion criteria. Identifying molecular genetic markers for why some students do not respond to evidence-based instruction early in literacy learning, which has been shown to be effective for many students, is relevant to understanding why, without blaming teachers, students, or parents, and modifying instructional programs to help them overcome their learning struggles.

Although this study was primarily of European Americans, in the authors’ research and clinical experience, the phenotype measures used to identify profiles [patterns] at the phenotype level based on inclusion criteria described in the methods have been found to identify dysgraphia, dyslexia, and OWL LD across racial groups. Likewise, children of all races have been shown to respond to both early intervention in literacy skills and intervention for persisting reading, writing, and oral language SLDs in the later grades. It may be that the profile of phenotypes and developmental histories associated with different SLDs interfering with language learning is more important than race in identifying hallmark phenotypes and their relationships with alleles for language learning; but further research on this genetically and educationally significant issue is warranted. That is not to negate that race is relevant to other conditions that are related to genetics.

### Next steps and relevance to the epigenetics of response to intervention [RTI]

Identifying hallmark allele-phenotype relationships can inform future research on RTI, which should include a focus on the epigenetics of response to instruction, both early in literacy acquisition and during middle childhood and early adolescence. It is likely that genotypes inherited at birth do not comprise the entirety of the genetic influence at the behavioral level during RTI; and the behavioral expression of underlying genetic markers may change across development due to both molecular markers of genetics variations and changes in the nature of curriculum requirements. Studying molecular epigenetic factors may also reveal mechanisms impeding learning, such as nutritional deficiencies or developmental disabilities or brain, which could be subject to targeted interventions to support more effective learning.

### Implications for translation science of genetics research to practice

#### Educating policy makers:

Current educational policy tends to blame teachers if students do not achieve a criterion score assumed to be an index of meeting common core standards. One of the important messages from genetics research is that variations in many oral and written language and language-related skills have genetics bases. Therefore, evidence-based policy and regulations should not blame and punish teachers when students with evidence-based SLD diagnoses do not achieve the criterion score in skills related to their diagnosed SLDs. Nor should parents or students with SLDs be blamed. Rather grade-appropriate differential instruction should be offered that is yoked to the nature of the diagnostic profile and its hallmark phenotypes and their associations with alleles. Response to that instruction should be monitored to evaluate whether the student’s learning improves, or if it does not, further modify the specially designed instruction until learning begins to improve. This approach to personalized education is grounded in a no-fault approach that recognizes the genetic variation accounting for individual as well as developmental differences in oral and written language learners [21]. Also, identifying molecular genetic markers does not mean it is impossible to overcome the language learning problems. Rather they provide explanation why some students struggle longer and may need instruction that is more carefully yoked to their learning profiles.

#### Explaining SLDs to members of interdisciplinary teams in schools:

Preservice training programs for professionals in different disciplines who work on interdisciplinary teams in schools should offer foundation courses to prepare future school professionals to become critical and effective consumers of the knowledge explosion in genetics and neuroscience that is relevant to understanding not only SLDs but also many other conditions in school-age populations [20] as well as typical learning. In addition, such foundational courses should also cover instructional science so that future educators are prepared for the ever growing body of relevant research on teaching language by ear, mouth, and hand and not just language by eye [reading] as reviewed by the National Reading Panel in 2000.

#### Explaining SLDs to parents of students with SLDs:

Such foundational knowledge would enable the educational professionals in schools to explain to parents the nature of their child’s SLDs. Parents of children with SLDs often seek not only instructional assistance for their children but also clear explanations, which are easy for them to understand, of what their children’s learning problems are and why they are having these problems. Knowledge of genetics research can inform those explanations parents are seeking. Knowledge of epigenetics research on RTI for SLDs in oral and/or written language, when available in the future, can offer hope that their child’s learning is likely to improve. The distinction between mediating molecular markers and gene sequencing inherited at birth may be crucial for communicating the potential plasticity in genetic-behavior relationships when the environment is tailored to a student’s personal learning profile.

#### Explaining SLDs to affected students:

Increasingly students with SLDs are learning about genetics in science courses at school and through the media, and if they participate in research on the genetics of SLDs, that SLDs have a genetic basis. They begin to wonder what it means to have an SLD, why they have genetic differences, and if there is hope they will overcome their genetic disorder. One challenge educational professionals face is reassuring the students with SLDs that there is a reason for the SLDs not related to their intelligence. Their struggles with oral and/or written language learning are not a sign that they are not smart. Yet blaming genes [or brain] for the learning struggles can also be personally alarming. Another challenge educational professionals face is reassuring students with SLDs, which have a genetic and brain basis, that they have relative strengths [and often talents] despite the SLD. Most importantly there is the challenge of convincing students with SLDs there is hope that, with specialized instruction, they can become successful readers and writers. Epigenetics research that informs knowledge of genetic-behavioral relationships before and after specialized, individualized instruction tailored to the nature of an individual’s SLD is the next frontier. Results of such epigenetics research will be relevant to reassuring affected students that there is hope that they can become successful oral and/or written language learners.

## Conclusions

Our study is consistent with the consensus that the genetic bases for learning oral and written language are complex. The phenotypes are also complex and may express differently at various stages of development, beginning in the preschool years, or early childhood and in some cases even persisting in middle childhood and adolescence during the school years. However, just because an SLD has a genetic basis does not mean it is not treatable. Research on the allele associations with hallmark phenotypes for different SLDs can inform future research on genetic mediators of the brain’s government of the complex reading and writing systems before and/or after instruction or possible epigenetic changes that may occur not only in response to maternal nurturing in the home [66], but also in response to instruction by educational professionals during the school years. Such studies would also have important applications for translation science of genetic-brain relationships during middle childhood and early adolescence for individuals who do and do not have persisting SLDs such as dysgraphia, dyslexia, and OWL LD.

## Figures and Tables

**Table 1.** Means (M) and Standard Deviations (SD) for each diagnostic group on each measure in assessment battery.

	Control group		Dysgraphia group		Dyslexia group		OWL LD group	
	M	SD	M	SD	M	SD	M	SD
**Alph 15^a^**	−.071	0.74	−1.54	0.66	−1.28	0.77	−1.44	0.70
**Copy Best^b^**	12.62	2.36	8.67	3.32	8.79	3.25	9.53	3.48
**Silent Word Reading^c^**	100.11	9.37	100.55	12.67	91.61	8.24	84.50	8.92
**Word Spelling^d^**	10.15	2.38	9.67	3.24	6.77	2.19	7.08	2.75
**Oral Compre^e^**	111.23	10.70	115.00	12.01	113.15	8.67	94.33	13.95
**Oral Sentences^f^**	11.28	2.78	10.05	3.20	11.65	2.36	5.60	2.38
**Verbal Compre^g^**	111.0	12.23	110.24	16.47	113.18	11.01	89.40	14.39

^f^Notes.

a Rapid Automatic Letter Writing

b *DASH Copy Sentences*—Best Handwriting Instructions

c TOSWRF Test of Silent Word Reading Fluency

d TOC Letter-Choice (add letter to create word-specific spelling)

e WJ3 Oral Comprehension

f CELF 4 Formulated Sentences

g *WISC IV Verbal Comprehension Index* See Methods in Text for information about each measure.

## References

[1] Astrom R, Wadsworth S, Olson R, Willicut E, DeFries J (2012) Genetic and environmental etiologies of reading difficulties: DeFries-Fulker analysis of reading performance data from twin pairs and their nontwin siblings. Learning and Individual Differences 22: 365–369. [Crossref] 22927712 10.1016/j.lindif.2012.01.011 PMC3423974

[2] Samuelsson S, Byrne B, Olson R, Hulslander J, Wadsworth S, (2008) Response to early literacy instruction in the United States, Australia, and Scandinavia: A behavioral- genetic analysis. Learning and Individual Differences 18: 289–295. [Crossref] 19122888 10.1016/j.lindif.2008.03.004 PMC2570222

[3] Smith SD, Kimberling WJ, Pennington BF, Lubs HA (1983) Specific reading 1800 disability: identification of an inherited form through linkage analysis. Science 219: 1345–1347. [Crossref] 6828864 10.1126/science.6828864

[4] Raskind W, Peters B, Richards T, Eckert M, Berninger V (2012) The Genetics of reading disabilities: From phenotype to candidate genes. Frontiers in Psychology 3: 601. [Crossref] 23308072 10.3389/fpsyg.2012.00601 PMC3538356

[5] Naidoo N, Pawitan Y, Soong R, Cooper DN, Ku CS (2011) Human genetics and genomics a decade after the release of the draft sequence of the human genome. Human Genomics 5: 577–622. [Crossref] 22155605 10.1186/1479-7364-5-6-577 PMC3525251

[6] Catts HW, Adlof SM, Hogan TP, Ellis Weismer S (2005) Are specific language impairment and dyslexia distinct disorders? Journal of Speech Language, and Hearing Research 48: 1378–1396. [Crossref] 10.1044/1092-4388(2005/096) PMC2853030 16478378

[7] Sanders E, Berninger V, Abbott R (2017) Sequential prediction of literacy achievement for specific learning disabilities contrasting in impaired levels of language in grades 4 to 9. Journal of Learning Disabilities. 10.1177/0022219417691048 PMC5538955 28199175

[8] Berninger V, Richards T, Abbott R (2015) Differential diagnosis of dysgraphia, dyslexia, and OWL LD: Behavioral and neuroimaging evidence. Reading and Writing. An Interdisciplinary Journal 28: 1119–1153. [Crossref] 10.1007/s11145-015-9565-0 PMC4553247 26336330

[9] Richards TL, Grabowksi T, Askren K, Boord P, Yagle K, Mestre Z, (2015) Contrasting brain patterns of writing-related DTI parameters, fMRI connectivity, and DTI-fMRI connectivity correlations in children with and without dysgraphia or dyslexia. Neuroimage Clin 8: 408–421. [Crossref] 26106566 10.1016/j.nicl.2015.03.018 PMC4473717

[10] Richards T, Nagy W, Abbott R, Berninger V (2016) Brain connectivity associated with cascading levels of language. Journal of Systems and Integrative Neuroscience (JSIN) 2: 219–229. 10.15761/JSIN.1000139 PMC5261811 28127444

[11] Yagle K, Richards T, Askren K, Mestre Z, Beers S, Abbott R, (2017) Relationships between eye movements during sentence reading comprehension, word spelling and reading, and DTI and fMRI connectivity in students with and without dysgraphia or dyslexia. Journal of Systems and Integrated Neuroscience 3(1): 1–11. 10.15761/JSIN.1000150 PMC5604484 28936361

[12] Cassiday L (2009) Mapping the epigenome. New tools chart chemical modifications of DNA and its packaging proteins. pp. 11–16. www.gen.on-line.org

[13] National Reading Panel (U.S.), National Institute of Child Health and Human Development (U.S.) (2000) Report of the National Reading Panel: Teaching children to read: An evidence-based assessment of the scientific research literature on reading and its implications for reading instruction: reports of the subgroups. Washington, D.C.: National Institute of Child Health and Human Development, National Institutes of Health

[14] Bishop DV (2015) The interface between genetics and psychology: lessons from developmental dyslexia. Proceedings Biological Science 282: 20143139. [Crossref] 10.1098/rspb.2014.3139 PMC4426619 25854887

[15] Peterson RL, Pennington BF (2015) Developmental dyslexia. Annual Review Clinical Psychology 11: 283–307. [Crossref] 10.1146/annurev-clinpsy-032814-112842 25594880

[16] Katusic SK, Colligan RC, Barbaresi WJ, Schaid DJ, Jacobsen SJ (2001) Incidence of reading disability in a population-based birth cohort, 1976–1982, Rochester, Minnesota. Mayo Clinic Proceedings 76: 1081–92. [Crossref] 11702896 10.4065/76.11.1081

[17] Katusic SK, Colligan RC, Weaver AL, Barbaresi WJ (2009) The forgotten learning disability - Epidemiology of written language disorder in a population-based birth cohort (1976–1982), Rochester, Minnesota Pediatrics 123: 1306–13. [Crossref] 10.1542/peds.2008-2098 PMC2923476 19403496

[18] Stoeckel RE, Colligan RC, Barbaresi WJ, Weaver AL, Killian JM, (2013) Early speech-language impairment and risk for written language disorder: a population-based study. Journal Developmental Behavior Pediatrics 34: 38–44. [Crossref] 10.1097/DBP.0b013e31827ba22a PMC3546529 23275057

[19] Wang Y, Paramasivam M, Thomas A, Bai J, Kaminen-Ahola N, (2006) DYX1C1 functions in neuronal migration in developing neocortex. Neuroscience 143: 515–522. [Crossref] 16989952 10.1016/j.neuroscience.2006.08.022

[20] Batshaw M, Roizen N, Lotrecchiano G (2013) Children with disabilities, 7th Ed. Baltimore, Md.: Paul H. Brookes

[21] Berninge VW (2015) Interdisciplinary frameworks for schools: Best professional practices for serving the needs of all students. Washington, DC: American Psychological Association.

[22] Lyytinen H, Aro M, Eklund K, Erskine J, Guttorm T, (2004) The development of children at familial risk for dyslexia: birth to early school age. Ann Dyslexia 54: 184–220. [Crossref] 15741935 10.1007/s11881-004-0010-3

[23] Nelson NW (2010) Language and literacy Disorders: Infancy through adolescence. Boston, MA: Allyn & Bacon.

[24] Catts HW, Bridges MS, Little TD, Tomblin JB (2008) Reading achievement growth in children with language impairments. Journal of Speech- Language and Hearing Research 51: 1569–1579. [Crossref] 10.1044/1092-4388(2008/07-0259) PMC3763805 18695010

[25] Lovett M, Barron R, Frijters J (2013) Word identification difficulties in children and adolescents with reading disabilities: Intervention research findings In Swanson HL, Harris K, & Graham S (Eds.), Handbook of Learning Disabilities (2nd ed.) (pp. 329–359) New York, NY: Guilford Press.

[26] Niedo J, Lee YL, Breznitz Z, Berninger V (2014) Computerized silent reading rate and strategy instruction for fourth graders at risk in silent reading rate. Learning Disability Quarterly 37: 100–110. [Crossref] 24914248 10.1177/0731948713507263 PMC4047714

[27] Treiman R, Kessler B (2014) How children learn to write words. New York: Oxford University Press.

[28] Connelly V, Dockrell J, Barnett A (2012) Children challenged by writing due to language and motor difficulties In Berninger V (Ed.), Past, present, and future contributions of cognitive writing research to cognitive psychology (pp. 217–245) New York: Psychology Press /Taylor Francis Group.

[29] Ehri L (1980) The role of orthographic images in learning printed words In Kavanaugh JF, & Venezky R (Eds.), Orthographic reading and dyslexia (pp. 307–332) Baltimore, MD: University Park Press.

[30] Olson R, Forsberg H, Wise B, Rack J (1994) Measurement of word recognition, orthographic, and phonological skills In Lyon GR (Ed.), Frames of reference for the assessment of learning disabilities (pp. 243–277) Baltimore, Brooks.

[31] Cahill L, Tiberius C, Herring J (2013) PolyOrth: Orthography, phonology, and morphology in the inheritance lexicons. Written Language and Literacy 16: 146–185.

[32] Lefly DL, Pennington BF (1991) Spelling errors and reading fluency in compensated adult dyslexics. Ann Dyslexia 41: 141–162. [Crossref] 24233762 10.1007/BF02648083

[33] Scott CM (2011) Assessment of language and literacy: A process of hypothesis testing. Topics in Language Disorders 31: 24–39.

[34] Bishop DVM (2009) Specific language impairment as a language learning disability. Child Language Teaching and Therapy 25: 163–165.

[35] Bishop DVM, Snowling MJ (2004) Developmental dyslexia and specific language impairment: Same or different? Psychological Bulletin 130: 858–886. [Crossref] 15535741 10.1037/0033-2909.130.6.858

[36] Silliman ER, Mody M (2008) Individual differences in oral language and reading: It’s a matter of individual differences In Mody M, & Silliman ER (Eds.), Brain, behavior, and learning in language and reading disorders (pp. 349–386) New York: Guilford.

[37] Silliman E, Berninger V (2011) Cross-disciplinary dialogue about the nature of oral and written language problems in the context of developmental, academic, and phenotypic profiles. Topics in Language Disorders 31: 6–23.

[38] Chomsky N (1965) Aspects of the theory of syntax. MIT Press.

[39] Darki F, Peyrard-Janvid M, Matsson H, Kere J, Klingberg T (2014) DCDC2 polymorphism is associated with left temporoparietal gray and white matter structures during development. Journal of Neuroscience 34: 14455–14462. 25339756 10.1523/JNEUROSCI.1216-14.2014 PMC6608392

[40] Ellis EM, Thal DJ (2008) Early language delay and risk for language impairment. Perspectives on Language Learning and Education 15: 93–100.

[41] Paul R, Murray C, Clancy K, Andrews D (1997) Reading and metaphonological outcomes in late talkers. J Speech Lang Hear Res 40: 1037–1047. [Crossref] 9328875 10.1044/jslhr.4005.1037

[42] Berninger V, Richards T (2010) Inter-relationships among behavioral markers, genes, brain, and treatment in dyslexia and dysgraphia. Future Neurology 5: 597–617. [Crossref] 20953351 10.2217/fnl.10.22 PMC2953808

[43] Shriffin R, Schneider W (1977) Controlled and automatic processing II: Perceptual learning, automatic attending, and a general theory. Psychological Review 84: 70–120.

[44] Berninger V, Nielsen K, Abbott R, Wijsman E, Raskind W (2008) Writing problems in developmental dyslexia: Under-recognized and under-treated. Journal of School Psychology 46: 1–21. [Crossref] 18438452 10.1016/j.jsp.2006.11.008 PMC2344144

[45] Barnett A, Henderson L, Scheib B, Schulz C (2007) Detailed Assessment of Speed of Handwriting (DASH) Copy Best and Fast. London: Pearson.

[46] Mather N, Hammill D, Allen E, Roberts R (2004) Test of Silent Word Reading Fluency TOSWRF. Austin, TX: Pro-Ed.

[47] Mather N, Roberts R, Hammill D, Allen E (2008) Test of Orthographic Competence (TOC) Austin, TX: Pro-Ed.

[48] Woodcock R, McGrew K, Mather N (2001) Woodcock-Johnson III Achievement Battery. Itasca, IL: Riverside.

[49] Semel E, Wiig EH, Secord WA (2003) Clinical Evaluations of Language

[50] Wechsler D (2003) Wechsler intelligence scale for children, 4th edition (WISC-IV) San Antonio, TX: The Psychological Corporation.

[51] Berninger V, Abbott R (2013) Children with dyslexia who are and are not gifted in verbal reasoning. Gifted Child Quarterly 57: 223–233. 10.1177/0016986213500342 PMC3829472 24249873

[52] van Viersen S, Kroesbergen EH, Slot EM, de Bree EH (2016) High Reading Skills Mask Dyslexia in Gifted Children. J Learn Disabil 49: 189–199. [Crossref] 24935885 10.1177/0022219414538517

[53] Taipale M, Kaminen N, Nopola- Hemmi J, Haltia T, Myllyluoma B, (2003a) A candidate gene for developmental dyslexia encodes a nuclear tetra- tricopeptide repeat domain protein dynamically regulated in brain. Proceedings National Academy of Sciences USA 100: 11553–11558. [Crossref] 10.1073/pnas.1833911100 PMC208796 12954984

[54] Tapia-Páez I, Tammimies K, Massinen S, Roy AL, Kere J (2008) The complex of TFII-I, PARP1, and SFPQ proteins regulates the DYX1C1gene implicated in neuronal migration and dyslexia. Federation of American Societies for Experimental Biology Journal 22: 3001–3009. 18445785 10.1096/fj.07-104455 PMC2493457

[55] Marino C, Citterio A, Giorda R, Facoetti A, Menozzi G, (2007) Association of short-term memory with a variant within DYX1C1 in developmental dyslexia. Genes Brain Behav 6: 640–646. [Crossref] 17309662 10.1111/j.1601-183X.2006.00291.x

[56] Tammimies K, Tapia-Paez I, Ruegg J, Rosin G, Kere J, (2012) The rs3743205 SNP is important for the regulation of the dyslexia candidate gene DYX1C1 by estrogen receptor beta and DNA methylation. Molecular Endocrinology 26: 619–629 22383464 10.1210/me.2011-1376 PMC5417142

[57] Darki F, Peyrard-Janvid M, Matsson H, Kere J, Klingberg T (2012) Three dyslexia susceptibility genes, DYX1C1, DCDC2, and KIAA0319, affect temporoparietal white matter structure. Biological Psychiatry 72: 671–676. 22683091 10.1016/j.biopsych.2012.05.008

[58] Bates TC, Lind PA, Luciano M, Montgomery GW, Martin NG, (2010) Dyslexia and DYX1C1: Deficits in reading and spelling associated with a missense mutation. Molecular Psychiatry 15: 1190–1196. [Crossref] 19901951 10.1038/mp.2009.120

[59] Bates TC, Luciano M, Medland SE, Montgomery GW, Wright MJ, (2011) Genetic variance in a component of the language acquisition device: ROBO1 polymorphisms associated with phonological buffer deficits. Behavior Genetics 41: 50–57. [Crossref] 20949370 10.1007/s10519-010-9402-9

[60] Schumacher J, Anthoni H, Dahdouh F, König IR, Hillmer AM, (2006) Strong genetic evidence of DCDC2 as a susceptibility gene for dyslexia. Am J Hum Genet 78: 52–62. [Crossref] 16385449 10.1086/498992 PMC1380223

[61] Wilcke A, Weissfuss J, Kirsten H, Wolfram G, Boltze J, (2009) The role of gene DCDC2 in German dyslexics. Ann Dyslexia 59: 1–11. [Crossref] 19238550 10.1007/s11881-008-0020-7

[62] Dennis MY, Paracchini S, Scerri TS, Prokunina-Olsson L, Knight JC, (2009) A common variant associated with dyslexia reduces expression of the KIAA0319 gene. PLoS Genetics 5: e1000436. 19325871 10.1371/journal.pgen.1000436 PMC2653637

[63] Curran S, Mill J, Sham P, Rijsdijk F, Marusic K, (2001) QTL association analysis of the DRD4 exon 3 VNTR polymorphism in a population sample of children screened with a parent rating scale for ADHD symptoms. American Journal of Medical Genetics 105: 387–393. 11378855 10.1002/ajmg.1366

[64] Vandenbergh DJ, Persico AM, Hawkins AL, Griffin CA, Li X, (1992) Human dopamine transporter gene (DAT1) maps to chromosome 5p15.3 and displays a VNTR. Genomics 14:1104–1106. [Crossref] 1478653 10.1016/s0888-7543(05)80138-7

[65] Hasler R, Salzmann A, Bolzan T, Zimmermann J, Baud P, (2015) DAT1 and DRD4 genes involved in key dimensions of adult ADHD. Neurol Sci 36: 861–869. [Crossref] 25555995 10.1007/s10072-014-2051-7

[66] Meaney MJ (2010) Epigenetics and the biological definition of gene x environment interactions. Child Dev 81: 41–79. [Crossref] 20331654 10.1111/j.1467-8624.2009.01381.x

